# Levels of Physical Fitness and Weight Status in Children and Adolescents: A Comparison between China and Japan

**DOI:** 10.3390/ijerph17249569

**Published:** 2020-12-21

**Authors:** Yuqiang Li, Feng Zhang, Qi Chen, Xiaojian Yin, Cunjian Bi, Xiaofang Yang, Yi Sun, Ming Li, Ting Zhang, Yuan Liu, Tao Chen, Akira Suzuki, Satoshi Haneda

**Affiliations:** 1 Key Laboratory of Adolescent Health Assessment and Exercise Intervention of the Ministry of Education, East China Normal University, Shanghai 200241, China; yqli@tyxx.ecnu.edu.cn (Y.L.); fzhang1988@126.com (F.Z.); 18721167130@163.com (Q.C.); cjbi1985@126.com (C.B.); fangxiaoyang2004@126.com (X.Y.); sunyi0084@163.com (Y.S.); liming23416@163.com (M.L.); noway1982@163.com (T.Z.); yliu0809@163.com (Y.L.); 2 College of Physical Education and Health, East China Normal University, Shanghai 200241, China; 3 College of Economics and Management, Shanghai Institute of Technology, Shanghai 201418, China; 4 Sport and Health Research Center, Department of Physical Education, Tongji University, Shanghai 200092, China; chentwhy@tongji.edu.cn; 5 Department of Sport Science, Daito Bunka University, Tokyo 355-8501, Japan; fwhz7271@yahoo.co.jp (A.S.); satohane@ic.daito.ac.jp (S.H.)

**Keywords:** physical fitness, child, adolescent, nutritional status, China, Japan

## Abstract

This study compared the physical fitness level and weight status of children and adolescents in China and Japan. Children and adolescents aged 7–18 years were recruited (China: *n* = 5660; Japan: *n* = 5660). Physical fitness was assessed using seven core items—grip strength, 30-s sit-ups, standing long jump, sit-and-reach, 20-s repeated straddling, 20-m shuttle run test, and 50-m dash. A physical fitness index (PFI) was calculated by adding all items’ *Z*-scores. We conducted comparisons of PFI and its distribution, each physical fitness item, and weight status for individuals from China and Japan across all ages. The PFI was lower in China than in Japan for all age groups, with an especially large difference at age 18 years for boys (a difference of 9.05) and girls (a difference of 9.10) (*p* < 0.001). The same result was seen for the seven items. The PFI distribution for children and adolescents was more disperse among those in Japan than among those in China. Obesity prevalence was 2.84 times higher in China than in Japan. An inverted U-shaped relationship was observed between physical fitness and nutritional status. Children and adolescents showed markedly lower physical fitness and higher obesity prevalence in China than in Japan.

## 1. Introduction

China and Japan, two close neighbors sharing multiple traits, together with South Korea, are often clustered into a single “Eastern Asian” cultural group and compared with Western cultures [1,2]. Grouping Eastern Asian cultures into one cluster might overlook important differences between countries. For example, although Japan and China have similar cultural origins, geographical climates, and Eastern Asian genetic characteristics, they differ in characteristics including nutrient intake [3], prevalence of overweight and obesity [4], physical activity levels [5,6], Human Development Index (HDI) [7], and urbanization [8], all of which are determinants of physical fitness.

Existing evidence suggests that physical fitness in childhood and adolescence is associated with total and abdominal adiposity, cardiovascular disease risk factors, skeletal health, psychological symptoms, and even academic performance [9]. In recent decades, a decline in physical fitness [10,11] and increased prevalence of overweight and obesity [12,13] are well documented in both China and Japan. According to a joint survey conducted by the two countries in 2016, the prevalence of overweight and obesity was 17.5% and 5.8% higher in China than in Japan among boys and girls [14]. A meta-analysis suggested a large decline (−5.4 per decade) in long-distance running performance from 1964 to 2009 among children in China, but only a small decline (−1.2 per decade) among children in Japan [15]. It was also reported that cardiopulmonary endurance was higher among children and adolescents in Japan than in China [16]. Furthermore, a recent study showed that adolescents in Japan outperformed those in China in terms of aerobic capacity and muscular endurance but that adolescents in China performed better than those in Japan in terms of muscular strength [17]. These previous studies compared only certain aspects of physical fitness. Comparisons of overall physical fitness between China and Japan are scarce.

In the present study, we calculated a physical fitness index (PFI) by combining seven core items (grip strength, 30-s sit-ups, sit-and-reach, standing long jump, 20-s repeated straddling, 50-m dash, and 20-m shuttle run test (SRT)) that were used to assess physical fitness at both the individual and population levels [18,19,20]. We aimed to compare the nutritional status and the mean and distribution of PFI overall and at specific percentiles of PFI among Chinese and Japanese children and adolescents.

## 2. Materials and Methods

### 2.1. Participants and Ethical Considerations

Considering geographical distribution, economic development, and urbanization, four regions were selected in China and Japan. Schools were chosen in each selected region. In China, urban/rural status was considered when choosing schools because of the wide gap in economic development between urban and rural areas, whereas this factor was not taken into account in Japan. The detailed sampling methods are reported elsewhere [16,21]. Individual participants without psychiatric or physical disorders were recruited from the selected schools using stratified random cluster sampling. Ultimately, data from 5660 students in China and 5660 students in Japan aged 7–18 years were included in the present study.

The study protocol was reviewed by the Medical Ethics Committee of East China Normal University and was approved after three revisions (approval number: HR2016/12055). The present study was conducted in accordance with the Declaration of Helsinki. Written informed consent was obtained from both the participants and their parents before the investigation. Participants’ names were coded to protect their privacy.

### 2.2. Procedure and Measurement

All study investigators, including physical education teachers and postgraduate students majoring in human sports science, were professionally trained before the investigation. After passing a training test, each investigator was responsible for only one test item to ensure the uniformity of the test standards.

For all children and adolescents, a portable stadiometer was used to measure height (to the nearest 0.1 cm), and a standardized scale was used to measure weight (0.1 kg). All participants were required to wear light clothing and to remove their shoes before these measurements were taken. Body mass index (BMI) was calculated as body weight (kg) divided by height (m^2^). All test procedures for the seven physical fitness items were conducted using the same types of instruments suggested by the new physical fitness test guidelines developed by the Japanese Sports Bureau of the Ministry of Education, Science, and Technology in 2008 [20,21].

### 2.3. Nutritional Status and PFI

Using the BMI-for-age *Z*-scores and height-for-age *Z*-scores, with the 2007 World Health Organization BMI standards as a reference, participants were classified as stunted (height-for-age *Z*-score < −2), underweight (BMI-for-age *Z*-score < −2), normal weight (BMI-for-age *Z*-score ≥ −2 and ≤ 1; height-for-age *Z*-score ≥ −2), overweight (BMI-for-age *Z*-score > 1 and ≤ 2 for), or obese (BMI-for-age *Z*-score > 2).

Physical fitness was assessed using seven core items—grip strength (reflecting upper-body muscle strength), 30-s sit-ups (reflecting abdominal muscle strength), standing long jump (reflecting lower-body muscle strength), sit-and-reach (reflecting hamstring and lower-back flexibility), 20-s repeated straddling (reflecting agility), 20-m SRT (reflecting aerobic fitness), and 50-m dash (reflecting explosive force and speed). To remove the unit of measurement and enable comparison between different items, the *Z*-score of each item was calculated, with the reference of the mean and standard deviation of Japanese children and adolescents aged 7–18 years, released by the Provincial Sports Bureau of Japan in 2014. The following formula was used to calculate these *Z*-scores:

Z score = (actual test value—mean of reference)/standard deviation of the reference.

The PFI was calculated by adding the *Z*-scores of six items and then subtracting the *Z*-score of the 50-m dash because a lower 50-m dash *Z*-score reflects better performance. The resulting value was used to evaluate the physical fitness of children and adolescents in China and Japan:

PFI = Z_grip strength_ + Z_30-s sit-ups_ + Z _standing long jump_ + Z_sit-and-reach_ + Z _20-s repeated straddling_ + Z _20-m SRT_ − Z _50-m dash._

### 2.4. Statistical Analyses

Characteristics were described using means (standard deviation (SD)), or frequencies (proportions). We estimated changing trends with age for the seven items and PFI by gender in both China and Japan. Differences in PFI between Chinese and Japanese boys and girls were assessed across PFI percentiles. The distributions of PFI means for each gender in both China and Japan were analyzed. The nutritional status (undernutrition, normal weight, or overnutrition) of children and adolescents in China and Japan were compared using the chi-square test. Given the difference in physical fitness between boys and girls, we stratified the following analyses by genders. A general linear model was used to examine the difference in PFI between countries, after adjusting for age groups. We also tested the interactions of age groups and countries to examine whether the difference between countries varied by age groups. The relationships between BMI-Z and PFI for different gender grouping were investigated using a nonlinear regression model (PFI = aBMI-Z^2^ + bBMI-Z + c; where a, b, and c were constants). The level of statistical significance was set at 0.05. All analyses were performed using IBM SPSS, Version 25.0 (IBM Inc., Armonk, NY, USA) and GraphPad Prism 8.0.2 (GraphPad Software, La Jolla, CA, USA).

## 3. Results

A total of 11,320 children and adolescents aged 7–18 years were included in the study. Overall, the sample had an equal boy-to-girl ratio, and there were comparable numbers of subjects in each age group in both China and Japan. In China, the selected children and adolescents were, on average, 3.4 cm taller than those in Japan. The percentage of children and adolescents with normal weight was 71.3% and 80.9% in China and Japan, respectively. The prevalence of overweight and obesity for children and adolescents were higher in China than in Japan. Particularly, the prevalence of obesity was 2.84 times higher in China than in Japan. Children and adolescents in Japan outperformed those in China in all physical fitness items, especially the 20-m SRT and 20-s repeated straddling, where children and adolescents in Japan scored approximately 50% higher, compared to those in China. There were also significant differences in physical fitness between Chinese and Japanese children and adolescents (*p* < 0.01). (Table 1).

**Figure 1:**
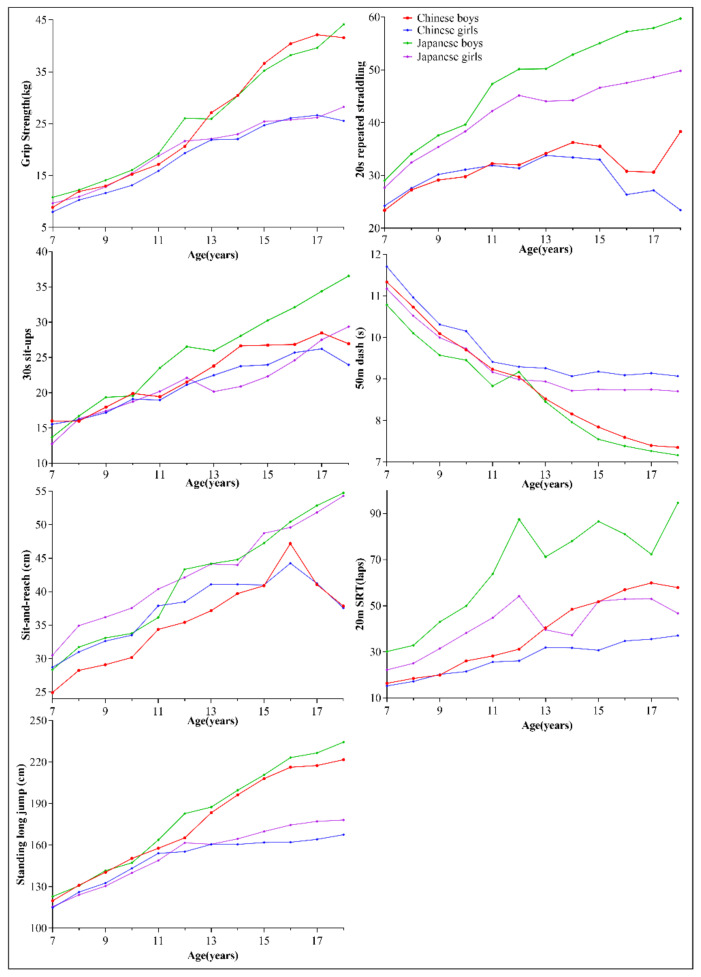
Age trends by gender of seven physical fitness items for children and adolescents aged 7–18 years in China and Japan.

Figure 1 shows the age trends of the seven physical fitness items for children and adolescents aged 7–18 years by gender in China and Japan. In general, with the exception of grip strength, Japan outperformed China on all items. For example, for the 20-m SRT, sit-and-reach, and the 20-s repeated straddling, Japanese boys had a better score than Chinese boys for all age groups. Japanese girls also performed better than Chinese girls on the 20-m SRT, sit-and-reach, 20-s repeated straddling, and 50-m dash for all age groups.

**Figure 2:**
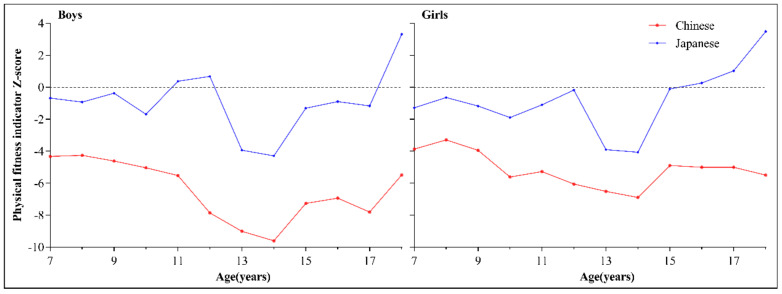
Age trends by gender of the physical fitness index for children and adolescents aged 7–18 years in China and Japan.

Gender stratified general linear model analysis indicated significant age (F_boys_ = 92.359, F_girls_ = 63.106, both *p* < 0.01), country (F_boys_ = 2973.392, F_girls_ = 2072.052, both *p* < 0.001), and age and country (F_boys_ = 26.413, F_girls_ = 34.199, both *p* < 0.01) interaction effects on PFI. Therefore, PFI comparison between Chinese and Japanese children and adolescents of the same age group was conducted; shown in Figure 2. Figure 2 shows the comparison of the mean PFI between China and Japan within the same age group stratified by gender. The PFI was clearly higher in Japan than in China for both boys and girls across all age groups. The largest differences in mean PFI between the two countries were observed at the age of 18 years for both boys (a difference of 9.05) and girls (a difference of 9.10), and the smallest differences occurred at the age of 10 years for boys (a difference of 3.59) and 14 years for girls (a difference of 3.41). All differences were statistically significant (*p* < 0.001).

**Figure 3:**
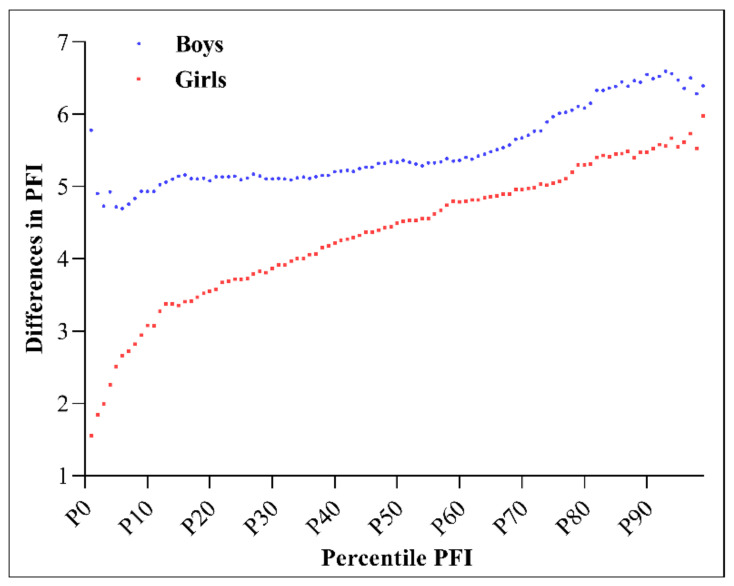
Physical fitness index (PFI) differences between China and Japan for each gender across PFI percentiles.

Figure 3 shows the differences in PFI between China and Japan for each gender across PFI percentiles. The China–Japan PFI differences were larger for boys than for girls, especially below the 50th percentile of PFI. For girls, this difference increased gradually with the PFI percentile, from 1.6 at the first percentile to 6.0 at the 99th percentile. For boys, this difference seemed to be relatively even across the PFI percentiles, ranging from 4.7 at the sixth percentile to 6.6 at the 93rd percentile.

**Figure 4:**
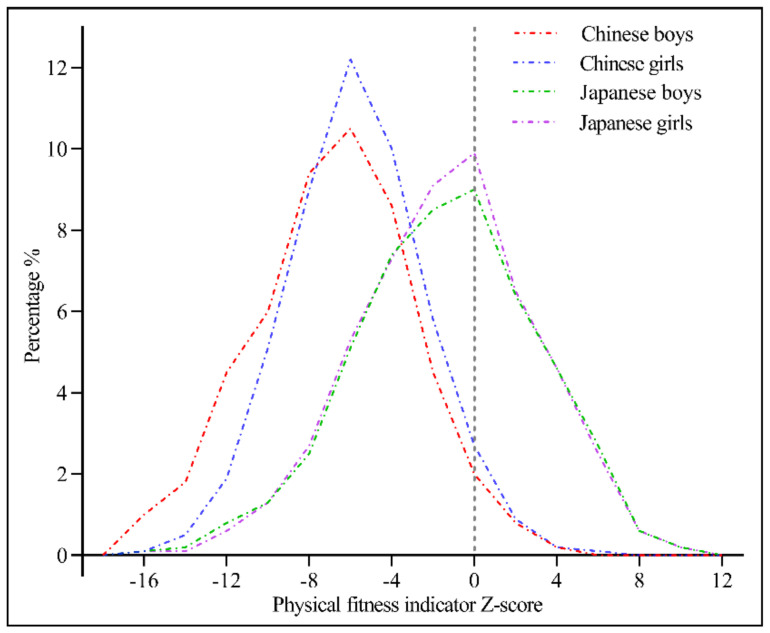
Physical fitness index distribution curves for children and adolescents by gender in China and Japan.

Figure 4 shows the distribution curves for PFI among children and adolescents of each gender in China and Japan. Compared with the distribution for China, the PFI distribution of children and adolescents in Japan was shifted to the right by about 6 units and was more disperse, for both boys and girls.

We compared the nutritional status between Chinese and Japanese children and adolescents, as shown in Table 2. Overall, the proportion of children with normal weight was significantly higher for Japan than for China, except among girls aged 13–18 years. The percentage of children suffering from undernutrition was highest among 10–12-year-old girls (8.0%) in China and among 13–15-year-old girls (9.3%) in Japan. The prevalence of overnutrition was significantly higher in China than in Japan for both boys and girls in every age group (*p* < 0.01). In particular, for 16–18-year-old boys, the prevalence of overnutrition in China (22.4%) was 2.8 times that in Japan (8.0%).

**Figure 5:**
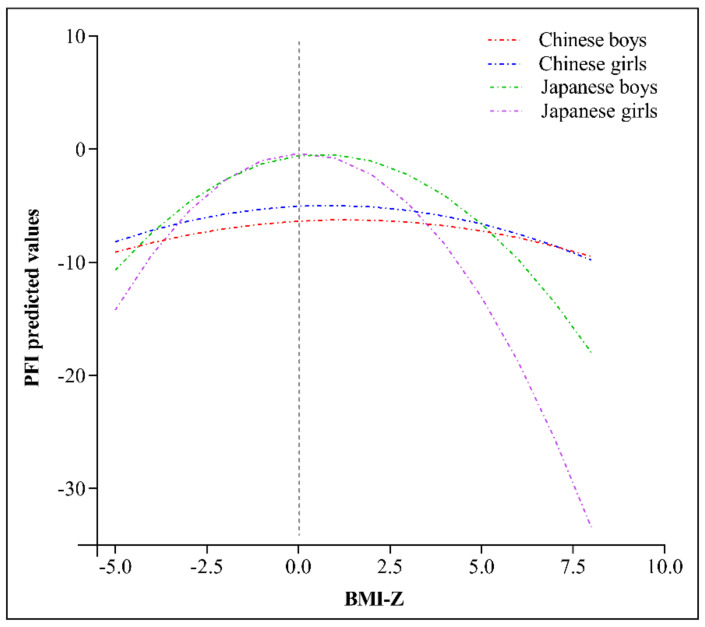
Relationship between PFI and BMI-Z score among boys and girls in China and Japan. Equations were listed as following: PFI (Chinese boys) = −0.072 × BMI-Z^2^ + 0.187 × BMI-Z − 6.363; PFI (Chinese girls) = −0.094 × BMI-Z^2^ + 0.157 × BMI-Z − 5.042; PFI (Japanese boys) = −0.323 × BMI-Z^2^ + 0.408 × BMI-Z − 0.576; PFI (Japanese girls) = −0.531 × BMI-Z^2^ + 0.114 × BMI-Z − 0.373.

Figure 5 shows the relationships (inverted U-shaped) between PFI and weight status of children and adolescents in both China and Japan. Among both Chinese and Japanese children and adolescents, the PFI value was the largest when the BMI–Z score was within the normal range. PFI declined when the BMI Z-score was higher or lower than the normal range. The quadratic coefficients of BMI-Z for PFI were: −0.072 and −0.323 in Chinese and Japanese boys; −0.094 and −0.531 in Chinese and Japanese girls.

## 4. Discussion

The present study compared PFI and weight status among children and adolescents aged 7–18 years in China and Japan, using an overall score of seven physical fitness items. We observed that PFI and each physical fitness item were higher in Japan than in China. The difference in PFI between the two countries was higher among boys than among girls across PFI percentiles, but especially at lower percentiles. The distribution of PFI was more disperse among children and adolescents in Japan than among those in China. There was an inverted U-shaped association between BMI Z-score and PFI in both countries. PFI value was the highest when the BMI Z-score was within the normal range.

An inverted U-shaped relationship between physical fitness and growth and nutritional status was previously reported for children and adolescents in both China and Japan [10,14], indicating that undernourished and overnourished students have lower physical fitness compared to their normal-weight counterparts. Overweight and obese South African adolescent girls were also reported to have lower cardiorespiratory fitness, decreased lower-body muscular strength, greater grip strength, and more joint hypermobility, compared to their peers with normal weight [22]. In the present study, we also observed a similar association between the BMI-Z score and PFI in boys and girls in both countries. China’s higher prevalence rates of overweight and obesity relative to Japan, might contribute to a poorer performance in physical fitness among Chinese children and adolescents. Higher BMI values correspond to higher resistance to overcome for physical fitness items such as 30-s sit-ups, standing long jump, 20-s repeated straddling, 50-m dash, and 20-m SRT, resulting in a poorer performance among individuals with higher BMI. Moreover, the association between BMI-Z score and PFI was stronger in Japan than in China for both boys and girls. Given limited evidence, further studies are needed to clarify this point.

According to the physical activity guidelines issued by the World Health Organization, physical fitness and health among children and young people are substantially enhanced by frequent physical activity, and at least 60 min of moderate-to-vigorous-intensity physical activity each day are recommended for children and adolescents [23]. It was reported that only 30% of Chinese children and adolescents met these recommended guidelines [19], compared with 44.7% in Japan [14]. Moreover, the report of 2018 Active Healthy Kids Global Alliance, initiated by Professor Mark Tremblay, suggested that Japan achieved the highest grades for active transportation (A‒) and physical fitness (A) worldwide [24], while grades for all areas representing physical activity in children and youth were particularly low (D‒F) in China [25]. Physical inactivity might be a factor contributing to children and adolescents in China having a lower PFI than their counterparts in Japan. In addition, compared to active young people, those who are physically inactive were found to have higher levels of body fat, which is related to poor physical fitness, as mentioned above [23].

Previous data from the Chinese Health and Nutrition Survey demonstrate that, from 1991 to 2011, young people in China aged 12–17 years changed their dietary habits from a carbohydrate-based diet to a Westernized high-fat, high-protein diet [26]. In addition, the percentage of Chinese children and adolescents consuming sugar-sweetened beverages increased from 73.58% in 2004 to 90.49% in 2011 [27]. In contrast, Japan introduced the “school nutrition lunch law” in 1954 to improve young people’s nutritional balance and encourage them to eat a healthy diet [28]. It is well documented that frequent breakfast eating is positively correlated with cardiorespiratory fitness among children and adolescents [29,30]. Existing evidence suggests that, among children and adolescents, 3.1% in China [31] and 2.3% in Japan [32] skip breakfast. These differences in dietary styles might contribute to the differences in physical fitness between China and Japan. Additionally, active commuting to and from school was reported to be significantly correlated with speed, agility, and muscle strength among students in Spain [33], England [34], and Colombia [35]. Sun et al. found that the percentages of students using active commuting methods (cycling or walking to and from school) in primary, middle, and high school were 55.8%, 54.6%, and 50.9%, respectively, in China [36], and 93%, 88%, and 68%, respectively, in Japan [37]. These differences in active commuting might be another reason for the lower physical fitness level of children and adolescents in China compared to those in Japan.

Socioeconomic status was reported to be positively associated with musculoskeletal and aerobic fitness [38,39,40]. Indicators such as the HDI and urbanization are often used to evaluate the social and economic status of a country. The HDI is a measure of human development with respect to the economy, education, and health; it effectively corrects the limitations of the traditional Gross Domestic Product Index and is widely used [41]. According to the United Nations Development Program [7], in 2018, Japan had a better HDI rank (19th) than China (85th), indicating that Japan had a higher level of development from the perspective of economy, education, and health. Urbanization refers to the proportion of people living in urban areas, reflecting the level of regional economic development [8]. According to a report released by the World Bank, the urbanization rates of China and Japan in 2014 were 54.26% and 91.30%, respectively [8]. The higher physical fitness of students in Japan than in China might also be explained by these national-level differences in socioeconomic development.

The main strength of the present article lies in the unified testing method used in both Japan and China. There are also several study limitations. First, this was a cross-sectional survey, and a cohort study is needed in the future to analyze the long-term trends in physical fitness among children and adolescents in China and Japan. Second, physical fitness is a comprehensive health indicator, and exactly which items should be included in a measure of physical fitness remains unclear. We used seven items (grip strength, 30-s sit-ups, sit-and-reach, standing long jump, 20-s repeated straddling, 50-m dash, and 20-m SRT) to calculate the PFI. Although this conclusion was not verified, Dong et al. [10] assessed the consistency of results across six consecutive national surveys and suggested that a comprehensive PFI captured the physical capabilities of children and adolescents. The approach to PFI adopted in the present study was used in several previous studies [10,42]. Third, we did not collect data on other factors, such as dietary intake, physical activity, and family environment, which might have substantial effects on the physical fitness and nutritional status of children and adolescents in China and Japan.

## 5. Conclusions

In conclusion, this study compared PFI and weight status between Chinese and Japanese children and adolescents, revealing large gaps between the two countries, especially in terms of agility and aerobic fitness. Further studies are needed to identify whether the determinants of physical fitness are different between the two countries, which might facilitate the development of efficient intervention strategies to improve and enhance physical fitness in both countries.

## Figures and Tables

**Table 1:** Basic characteristics, nutritional status, and physical fitness status among Chinese and Japanese children and adolescents aged 7–18 years.

	China			Japan			*t*/*χ*^2^	*p*-Values
	Boys	Girls	Total	Boys	Girls	Total		
Sample size (%)	2850 (50.4)	2810 (49.6)	5660	2847 (50.3)	2813 (49.7)	5660	/	/
Age groups (years)								
7–9 (%)	731 (50.7)	712 (49.3)	1443	696 (50.4)	684 (49.6)	1380	/	/
10–12 (%)	711 (50.1)	709 (49.9)	1420	695 (49.9)	697 (50.1)	1392	/	/
13–15 (%)	704 (50.9)	680 (49.1)	1384	734 (50.8)	711 (49.2)	1445	/	/
16–18 (%)	704 (49.8)	709 (50.2)	1413	722 (50.0)	721 (50.0)	1443	/	/
Nutritional status								
Height (cm, SD)	154.3 (19.2)	149.6 (14.9)	152 (17.4)	151.1 (18.2)	146.1 (13.8)	148.6 (16.3)	10.728	<0.01
Weight (kg, SD)	48.3 (18.2)	43.1 (13.7)	45.7 (16.4)	43.8 (15.1)	40.2 (11.5)	42.0 (13.6)	13.065	<0.01
BMI (kg/m^2^, SD)	19.5 (4.0)	18.7 (3.4)	19.1 (3.7)	18.5 (2.9)	18.4 (2.8)	18.4 (2.9)	11.202	<0.01
Stunting (%)	72 (2.5)	75 (2.7)	147 (2.6)	143 (5.0)	179 (6.4)	322 (5.7)	68.12	<0.01
Thinness (%)	56 (2.0)	31 (1.1)	87 (1.5)	11 (0.4)	11 (0.4)	22 (0.4)	39.14	<0.01
Normal weight (%)	1835 (64.4)	2203 (78.4)	4038 (71.3)	2247 (78.9)	2331 (82.9)	4578 (80.9)	141.68	<0.01
Overweight (%)	384 (13.5)	289 (10.3)	673 (11.9)	274 (9.6)	223 (7.9)	497 (8.8)	29.53	<0.01
Obese (%)	518 (18.2)	221 (7.9)	739 (13.1)	179 (6.3)	80 (2.8)	259 (4.6)	253.18	<0.01
Physical fitness items								
Grip strength (kg, SD)	25.2 (13.2)	18.7 (7.5)	22.0 (11.2)	26.1 (12.5)	20.1 (7.3)	23.1 (10.7)	−5.343	<0.01
30-s sit-ups (SD)	22.5 (6.5)	21.1 (5.7)	21.8 (6.1)	25.6 (8.8)	21.1 (7.1)	23.4 (8.3)	−11.686	<0.01
Standing long jump (cm, SD)	175 (39.7)	150.1 (22.1)	162.7 (34.5)	181.4 (42.8)	154.2 (29.0)	167.9 (39)	−7.513	<0.01
Sit-and-reach (cm, SD)	35.4 (11.2)	37.4 (10.5)	36.4 (10.9)	41.8 (12.2)	42.9 (11.0)	42.4 (11.6)	−28.358	<0.01
20-s repeated straddling (SD)	31.5 (9.8)	29.4 (9.0)	30.5 (9.4)	47.7 (11.3)	42 (8.4)	44.8 (10.3)	−77.151	<0.01
50-m dash (s, SD)	8.9 (1.5)	9.7 (1.1)	9.3 (1.4)	8.6 (1.4)	9.3 (1.1)	9.0 (1.3)	11.814	<0.01
20-m SRT (laps, SD)	37.7 (21.6)	27.3 (11.8)	32.5 (18.2)	66.2 (24.8)	41.7 (14.2)	54.0 (23.6)	−54.274	<0.01

Note: Data are presented as n (%) or mean (SD), unless specified otherwise. 30-s sit-ups and 20-s repeated straddling were assessed by observing the number of repetitions. BMI = body mass index, PFI = physical fitness index, SD = standard deviation. T-tests and χ^2^ tests were compared between China and Japan.

**Table 2:** Comparison of nutritional status between Chinese and Japanese children and adolescents.

Age (y)	China				Japan				*χ* ^*2*-A^	*χ* ^*2*-B^	*χ* ^*2*-C^
	*N*	Under Nutrition *n* (%)	Normal *n* (%)	Overnutrition *n* (%)	*N*	Under Nutrition *n* (%)	Normal, *n* (%)	Overnutrition *n* (%)			
Boys											
7–9	731	30 (4.1)	439 (60.1)	262 (35.8)	696	32 (4.6)	549 (78.9)	115 (16.5)	0.21	59.32 ^b^	68.45 ^b^
10–12	711	48 (6.8)	390 (54.9)	273 (38.4)	695	31 (4.5)	484 (69.6)	180(25.9)	3.48	32.68 ^b^	25.14 ^b^
13–15	704	32 (4.5)	473 (67.2)	199 (28.3)	734	57 (7.8)	582 (79.3)	95 (12.9)	6.42 ^a^	26.94 ^b^	51.88 ^b^
16–18	704	13 (1.8)	533 (75.7)	158 (22.4)	722	32 (4.4)	632 (87.5)	58 (8.0)	7.80 ^b^	33.33 ^b^	57.59 ^b^
Girls											
7–9	712	22 (3.1)	523 (73.5)	167 (23.5)	684	34 (5.0)	553 (80.8)	97 (14.2)	3.21	10.79 ^b^	19.57 ^b^
10–12	709	57 (8.0)	518 (73.1)	134 (18.9)	697	39 (5.6)	565 (81.1)	93 (13.3)	3.30	12.72 ^b^	8.02 ^b^
13–15	680	11 (1.6)	541 (79.6)	128 (18.8)	711	66 (9.3)	566 (79.6)	79 (11.1)	39.05 ^b^	0.00	16.32 ^b^
16–18	709	11 (1.6)	621 (87.6)	77 (10.9)	721	51 (7.1)	647 (89.7)	23 (3.2)	26.28 ^b^	1.64	32.34 ^b^

Note: Undernutrition included stunting and thinness. Overnutrition included overweight and obesity. ^a^ < 0.05; ^b^ < 0.01. χ^2^-A represents the comparison of undernutrition between China and Japan; χ^2^-B represents the comparison of normal weight between China and Japan; χ^2^-C represents the comparison of overnutrition between China and Japan.

## References

[1] He B.M., Chen R., Sun T.Q., Yang Y., Zhang C.L., Ren S.C., Gao X., Sun Y.H. (2020). Prostate cancer risk prediction models in Eastern Asian populations: Current status, racial difference, and future directions. Asian J..

[2] Maruyama H., Ujiie T., Takai J., Takahama Y., Sakagami H., Shibayama M., Fukumoto M., Ninomiya K., Ah P.H., Feng X. (2015). Cultural Difference in Conflict Management Strategies of Children and Its Development: Comparing 3- and 5-Year-Olds across China, Japan, and Korea. Early Educ. Dev..

[3] Zhou B.F., Stamler J., Dennis B., Moag-Stahlberg A., Okuda N., Robertson C., Zhao L., Chan Q., Elliott P., INTERMAP Research Group (2003). Nutrient intakes of middle-aged men and women in China, Japan, United Kingdom, and United States in the late 1990s: The INTERMAP study. J. Hum. Hypertens..

[4] Noh J.W., Kim J., Yang Y., Park J., Cheon J., Kwon Y.D. (2017). Body mass index and self-rated health in East Asian countries: Comparison among South Korea, China, Japan, and Taiwan. PLoS ONE.

[5] Yamakita M., Sato M., Suzuki K., Ando D., Yamagata Z. (2018). Sex Differences in Birth Weight and Physical Activity in Japanese Schoolchildren. J. Epidemiol..

[6] Zhang Z., Zhang L., Li H. (2020). A meta-analysis of the physical activity status of children and adolescents in China. Chin. J. Sch. Health.

[7] United Nations Development Programme (2014). Human Development Reports. http://hdr.undp.org/en/data#.

[8] The World Bank Urban Population (% of Total). http://data.worldbank.org/indicator/SP.URB.TOTL.IN.ZS?display=default.

[9] Ortega F.B., Ruiz J.R., Castillo M.J., Sjostrom M. (2008). Physical fitness in childhood and adolescence: A powerful marker of health. Int. J. Obes. (Lond.).

[10] Dong Y., Lau P.W.C., Dong B., Zou Z., Yang Y., Wen B., Ma Y., Hu P., Song Y., Ma J. (2019). Trends in physical fitness, growth, and nutritional status of Chinese children and adolescents: A retrospective analysis of 1·5 million students from six successive national surveys between 1985 and 2014. Lancet Child Adolesc. Health.

[11] Ministry of Education, Culture, Sports, Science and Technology The Report of Survey on Physical Strength and Athletic Performance. http://www.mext.go.jp/a_menu/sports/kodomo/zencyo/1342657.htm.

[12] Guo Y., Yin X., Wu H., Chai X., Yang X. (2019). Trends in Overweight and Obesity among Children and Adolescents in China from 1991 to 2015: A Meta-Analysis. Int. J. Environ. Res. Public Health.

[13] Wakamatsu M., Sugawara Y., Zhang S., Tanji F., Tomata Y., Tsuji I. (2019). Weight change since age 20 and incident risk of obesity-related cancer in Japan: A pooled analysis of the Miyagi Cohort Study and the Ohsaki Cohort Study. Int. J. Cancer.

[14] Shao J., Sun Y., Yin X., Li Y., Akira S. (2019). Association between body mass index and physical fitness index in Chinese and Japanese children and adolescents. Chin. J. Sch. Health.

[15] Tomkinson G.R., Macfarlane D., Noi S., Kim D.Y., Wang Z.Z., Hong R. (2012). Temporal Changes in Long-Distance Running Performance of Asian Children between 1964 and 2009. Sports Med..

[16] Yang X., Yin X., Ji L., Song G., Wu H., Li Y., Wang G., Bi C., Sun Y., Li M. (2019). Differences in Cardiorespiratory Fitness between Chinese and Japanese Children and Adolescents. Int. J. Environ. Res. Public Health.

[17] Hui S.S., Zhang R., Suzuki K., Naito H., Balasekaran G., Song J.K., Park S.Y., Liou Y.M., Lu D., Poh B.K. (2020). Physical activity and health-related fitness in Asian adolescents: The Asia-fit study. J. Sports Sci..

[18] Bi C., Zhang F., Gu Y., Song Y., Cai X. (2020). Secular Trend in the Physical Fitness of Xinjiang Children and Adolescents between 1985 and 2014. Int. J. Environ. Res. Public Health.

[19] (2008). Japanese Sports Bureau of the Ministry of Education. https://www.mext.go.jp/sports/content/1408001_1.pdf.

[20] (2008). Japanese Sports Bureau of the Ministry of Education. https://www.mext.go.jp/sports/content/1408001_2.pdf.

[21] Yin X., Yang X., Ji L., Song G., Wu H., Li Y., Sun Y., Bi C., Li M., Zhang T. (2020). Comparison of growth and nutritional status of Chinese and Japanese children and adolescents. Ann. Hum. Biol..

[22] Bonney E., Ferguson G., Smits-Engelsman B. (2018). Relationship between Body Mass Index, Cardiorespiratory and Musculoskeletal Fitness among South African Adolescent Girls. Int. J. Environ. Res. Public Health.

[23] World Health Organization (2010). Global Recommendations on Physical Activity for Health.

[24] Report of 2018 Active Healthy Kids Global Alliance. https://www.activehealthykids.org/japan/.

[25] Report of 2018 Active Healthy Kids Global Alliance. https://www.activehealthykids.org/china/.

[26] Yu A.Y.L., Lopez-Olmedo N., Popkin B.M. (2018). Analysis of dietary trends in Chinese adolescents from 1991 to 2011. Asia Pac. J. Clin. Nutr..

[27] Jing F., Li Y., Fan C. (2018). Association between sweetened beverages consumption and obesity in Chinese children and adolescents. Prev. Med..

[28] Kohri T., Kaba N., Itoh T., Sasaki S. (2016). Effects of the National School Lunch Program on Bone Growth in Japanese Elementary School Children. J. Nutr. Sci. Vitaminol..

[29] Sandercock G.R.H., Voss C., Dye L. (2010). Associations between habitual school-day breakfast consumption, body mass index, physical activity and cardiorespiratory fitness in English schoolchildren. Eur. J. Clin. Nutr..

[30] David T., Julien A., Laurie I., Nordine L., Sebastien R., Eric D., Martine M., Pascale D. (2013). Are eating habits associated with physical fitness in primary school children?. Eat. Behav..

[31] Du W., Wang H., Wang D., Su C., Zhang J., Ouyang Y., Jia X., Huang F., Zhang B. (2016). Meal and snack consumption among Chinese children and adolescents in twelve provinces. J. Hyg. Res..

[32] Murakami K., Livingstone M.B.E., Fujiwara A., Sasaki S. (2018). Breakfast in Japan: Findings from the 2012 National Health and Nutrition Survey. Nutrients.

[33] Villa-Gonzalez E., Ruiz J.R., Chillon P. (2015). Associations between Active Commuting to School and Health-Related Physical Fitness in Spanish School-Aged Children: A Cross-Sectional Study. Int. J. Environ. Res. Public Health.

[34] Voss C., Sandercock G. (2010). Aerobic Fitness and Mode of Travel to School in English Schoolchildren. Med. Sci. Sports Exerc..

[35] Ramirez-Velez R., Garcia-Hermoso A., Agostinis-Sobrinho C., Mota J., Santos R., Enrique Correa-Bautista J., Constanza Amaya-Tambo D., Villa-Gonzalez E. (2017). Cycling to School and Body Composition, Physical Fitness, and Metabolic Syndrome in Children and Adolescents. J. Pediatrics.

[36] Sun Y., Liu Y., Tao F.B. (2015). Associations between Active Commuting to School, Body Fat, and Mental Well-being: Population-Based, Cross-Sectional Study in China. J. Adolesc. Health.

[37] Tanaka C., Tanaka S., Inoue S., Miyachi M., Suzuki K., Reilly J.J. (2016). Results from Japan’s 2016 Report Card on Physical Activity for Children and Youth. J. Phys. Act. Health.

[38] Wolfe A.M., Lee J.A., Laurson K.R. (2020). Socioeconomic status and physical fitness in youth: Findings from the NHANES National Youth Fitness Survey. J. Sports Sci..

[39] Pavon D.J., Ortega F.P., Ruiz J.R., Romero V.E., Artero E.G., Urdiales D.M., Martinez S.G., Rodriguez G.V., Manios Y., Beghin L. (2010). Socioeconomic status influences physical fitness in European adolescents independently of body fat and physical activity: The HELENA Study. Nutr. Hosp..

[40] Bowser J., Martinez-Donate A.P., Carrel A., Allen D.B., Moberg D.P. (2016). Disparities in Fitness and Physical Activity among Children. WMJ Off. Publ. State Med. Soc. Wis..

[41] Hu A., Shi Z., Tang X. (2018). The continues decreased and influencing factors of HDI difference in China. J. Xinjiang Norm. Univ. (Philos. Soc. Sci.).

[42] Huang Y.-C., Malina R.M. (2007). BMI and Health-Related Physical Fitness in Taiwanese Youth 9–18 Years. Med. Sci. Sports Exerc..

